# Association between hemoglobin glycation index and diabetic kidney disease in type 2 diabetes mellitus in China: A cross- sectional inpatient study

**DOI:** 10.3389/fendo.2023.1108061

**Published:** 2023-03-08

**Authors:** Sixu Xin, Xin Zhao, Jiaxiang Ding, Xiaomei Zhang

**Affiliations:** ^1^ Department of Endocrinology, Peking University International Hospital, Beijing, China; ^2^ Department of Nephrology, Peking University International Hospital, Beijing, China

**Keywords:** diabetes mellitus, type 2, hemoglobin glycation index, diabetic kidney disease, complication

## Abstract

**Objective:**

To investigate the association between Hemoglobin Glycation Index (HGI) and Diabetic Kidney Disease (DKD) in Chinese type 2 diabetic individuals and to construct a risk score based on HGI to predict a person’s risk of DKD.

**Methods:**

We retrospectively analyzed 1622 patients with type 2 diabetes mellitus (T2DM). HGI was obtained by calculating the fasting plasma glucose (FPG) level into the formula, and they were grouped into low HGI group (L-HGI), medium HGI group (H-HGI) and high HGI group (H-HGI) according to tri-sectional quantile of HGI. The occurrence of DKD was analyzed in patients with different levels of HGI. Multivariate logistics regression analysis was used to analyze the risk factors of DKD in patients with T2DM.

**Results:**

A total of 1622 patients with T2DM were enrolled in the study. Among them, 390 cases were DKD. The prevalence of DKD among the three groups was 16.6%, 24.2% and 31.3%. The difference was statistically significant (P = 0.000). There were significant differences in age (P=0.033), T2DM duration (P=0.005), systolic blood pressure (SBP) (P=0.003), glycosylated hemoglobin (HbA1c) (P=0.000), FPG (P=0.032), 2-hour postprandial plasma glucose (2h-PPG) (P=0.000), fasting C-peptide FCP (P=0.000), 2-hour postprandial C-peptide (2h-CP) (P=0.000), total cholesterol (TC) (P=0.003), low density lipoprotein cholesterol (LDL-C) (P=0.000), serum creatinine (sCr) (P=0.001), estimated glomerular filtration rate (eGFR) (P=0.000) among the three groups. Mantel-Haenszel chi-square test showed that there was a linear relationship between HGI and DKD (x2=177.469, p < 0.001). Pearson correlation analysis showed that with the increase of HGI level the prevalence of DKD was increasing (R= 0.445, P=0.000). It was indicated by univariate logistic regression analysis that individuals in H-HGI was more likely to develop DKD (OR: 2.283, 95% CI: 1.708~ 3.052) when compared with L-HGI. Adjusted to multiple factors, this trend still remained significant (OR: 2.660, 95% CI: 1.935~ 3.657). The combined DKD risk score based on HGI resulted in an area under the receiver operator characteristic curve (AUROC) of 0.702.

**Conclusions:**

High HGI is associated with an increased risk of DKD. DKD risk score may be used as one of the risk predictors of DKD in type 2 diabetic population.

## 1 Introduction

Diabetic kidney disease (DKD) is an important microvascular complication of diabetes and has become the main cause of chronic kidney disease (CKD) and end stage renal disease (ESRD) (1–3). Early detection of DKD and conducting the most effective targeted intervention are the key steps to offset the development of adverse clinical outcomes of diabetes mellitus (DM). Glycated hemoglobin (HbA1c) level is recommended as the gold standard method to evaluate glycemic control in DM patients. Despite the generally acknowledged role of HbA1c in the management of patients with diabetes, considerable differences in HbA1c exist even in patients with similar mean blood glucose (MBG) profiles (4, 5). Studies have shown that some individuals have persistently higher or lower HbA1c levels than expected. However, recent researches had shown that considerable biological variation of HbA1c was not only affected by blood glucose levels but also influenced by interindividual biological differences and environmental factor (6, 7). That means, even at the same blood glucose level, the level of HbA1c could be different. Therefore, solely relying on HbA1c level to evaluate the risk of DM is not suitable for all populations, which will produce a significant deviation. Hempe et al. (8) described this discrepancy by hemoglobin glycation index (HGI), which was calculated as the difference between an individual’s observed HbA1c and the estimated HbA1c.

HGI can identify people with HbA1c levels that are higher or lower than average compared to other people with the same blood glucose concentration (8, 9). It has been found that HGI could promote the development of some microvascular and macrovascular complications in DM patients (10). A recent meta-analysis showed that increased HbA1c variability was associated with increased risk of all-cause mortality, cardiovascular disease (CVD), renal disease, peripheral neuropathy in patients with type 2 diabetes mellitus (T2DM) (11). In the Diabetes Control and Complications Trial (DCCT), a high HGI at the baseline was a predictor of CKD and retinopathy in patients with T1DM after 7 years of follow-up (12). The individual variation in HbA1c observed in the DCCT was attributable to biological variation and not measurement error. In the Action in Diabetes and Vascular Disease: Preterax and Diamicron Modified Release Controlled Evaluation (ADVANCE) trial, a high HGI at the baseline predicted major microvascular events, which comprised new or worsened nephropathy or retinopathy in T2DM (13). Chih-Hung Lin et al. (14) found that HGI independently predicted renal function deterioration in patients with T2DM and a low CKD risk. Although cumulative evidence suggests a role for HGI in diabetes complications, Lachin et al. (15) had contradictory findings on reassessment of HGI for prediction of microvascular complications in the DCCT. They concluded that HGI was not a useful predictor for microvascular complications because it is not statistically independent of HbA1c.

Although the above-mentioned studies have shed light on the potential application of HGI in the management of diabetic complications, the evidence for HGI as a predictor of DKD remains unclear. Therefore, this study intends to analyze the relationship between HGI level and the risk of DKD in Chinese patients with T2DM and to construct a risk score to conveniently predict a person’s risk of DKD aiming to provide a new reference for clinical evaluation of diabetic complications.

## 2 Materials and methods

### 2.1 Participants

In this retrospective study, 1622 T2DM who were hospitalized in Peking University International Hospital endocrinology department from March 2015 to April 2021 were analyzed. Among them, 1016 (62.64%) were males and 606 (37.36%) were females, with an average age of (55.8 ± 13.47) years. The average duration of T2DM was 9.31 ± 7.73 years. All subjects met the T2DM diagnostic criteria of the World Health Organization (WHO) in 1999 (16). According to the diagnostic criteria of DKD (17), the subjects were divided into 1232 cases of non-DKD and 390 cases of DKD. The exclusion criteria included: (1) Other type of diabetes mellitus; (2) Acute complications of diabetes; (3) With primary renal parenchyma; (4) Recent urinary tract infection, taking drugs that affect renal function, etc.; (5) With severe anemia or blood loss; (6) Pregnant and lactating women; (7) Patients who were hospitalized for twice or more times.

### 2.2 Methods

#### 2.2.1 General conditions collected

All participants’ age, date of birth and diabetic duration (unit by year) were collected and recorded. All participants were asked to take off their shoes and socks and wear light and thin clothes, following which height (cm) and weight (kg) were measured with measuring instrument, and body mass index (BMI) was obtained according to the formula weight/height^2^ (kg/m^2^). Blood pressure including systolic blood pressure (SBP) and diastolic blood pressure (DBP) were measured in all participants.

#### 2.2.2 Laboratory measurement

All subjects were asked to fast for at least 8 hours, and venous blood samples were collected in the morning. Chemiluminescence method was then used to test blood glucose and blood lipid profile. Other biochemical indices were then determined. High-pressure liquid chromatography was used to test HbA1c level. The tests were carried out in the biochemical laboratory of Peking University International Hospital. Laboratory measurements included fasting plasma glucose (FPG), 2 hour postprandial plasma glucose (2h-PPG), fasting C-Peptide (FCP), 2 hour C-Peptide (2h-CP), HbA1c, low-density lipoprotein cholesterol (LDL-C), total cholesterol (TC), triglyceride (TG), high-density lipoprotein cholesterol (HDL-C), uric acid (UA), serum creatinine (sCr), alanine aminotransferase (ALT), aspartate aminotransferase (AST) and urinary microalbumin/creatinine ratio (UACR). The estimated glomerular filtration rate (eGFR) was estimated according to the sCr level.

#### 2.2.3 HGI calculation

**Figure 1:**
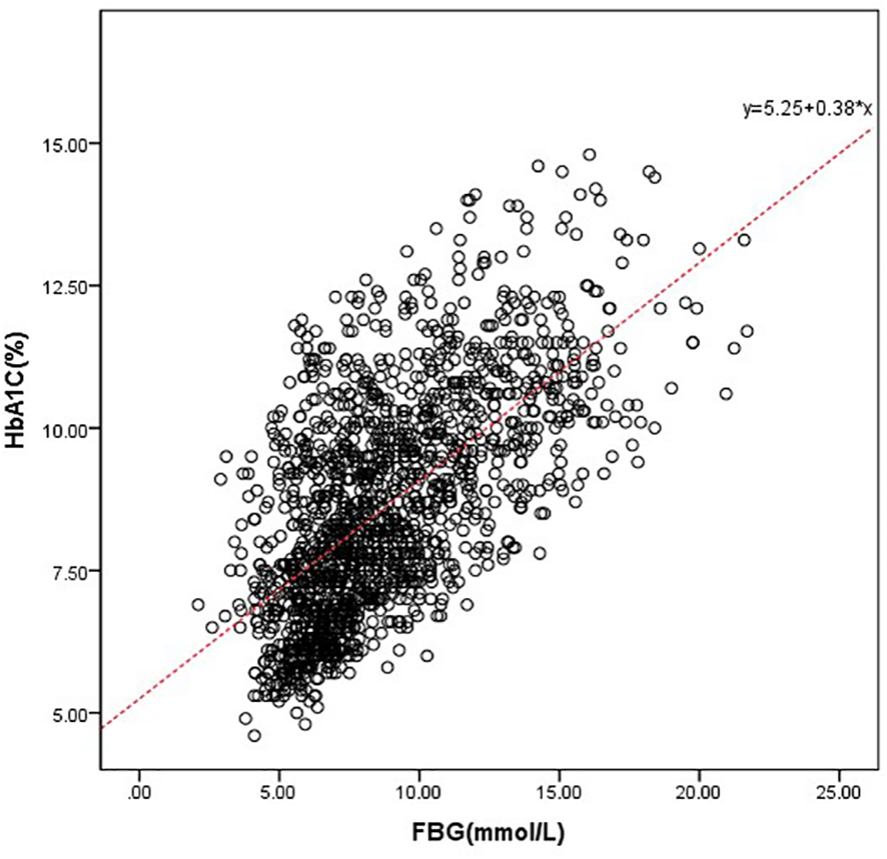
Correlation between HbA1c and FPG. We drove the predicted HbA1c, which was defined as follows: predict HbA1c= 5.249 + 0.383* FPG (r= 0.636 and p<0.001).

Taking the actual measured value of HbA1c as the dependent variable and FPG as the independent variable, a linear regression equation was established as follows: predict HbA1c= 5.249 + 0.383* FPG (r= 0.636 and p<0.001). Predicted HbA1c was then subtracted from the individual’s observed HbA1c to generate HGI (HGI = observed HbA1c – predicted HbA1c). HGI values were divided into three groups by using the tri quantile method: L-HGI, M-HGI and H-HGI (**Figure 1**).

### 2.3 Statistical methods

All data were processed by SPSS 22.0. Normal distribution data were shown as mean standard deviation ( ± s), and nonnormal distribution data were shown as mean median and quartile spacing. When quantitative data were normally distributed and variance was homogeneous, variance analysis was used for comparison among groups. When data were not normally distributed, variance analysis such as Kruskal Wallis test was used for comparison among multiple groups; qualitative data was expressed in percentage (%). Chi-square test was used to compare the qualitative data among the three groups. Pearson correlation analysis was used to analyze the correlation between HbA1c and FPG, and the regression equation was established accordingly. Logistic regression method was used for analysis of the main influencing factors of DKD with T2DM, and p< 0.05 was used for statistical significance. The AUROC was used to evaluate the sensitivity of the proposed risk score for the prediction of DKD.

## 3 Results

### 3.1 General characteristics among the 3 groups

**Figure 2:**
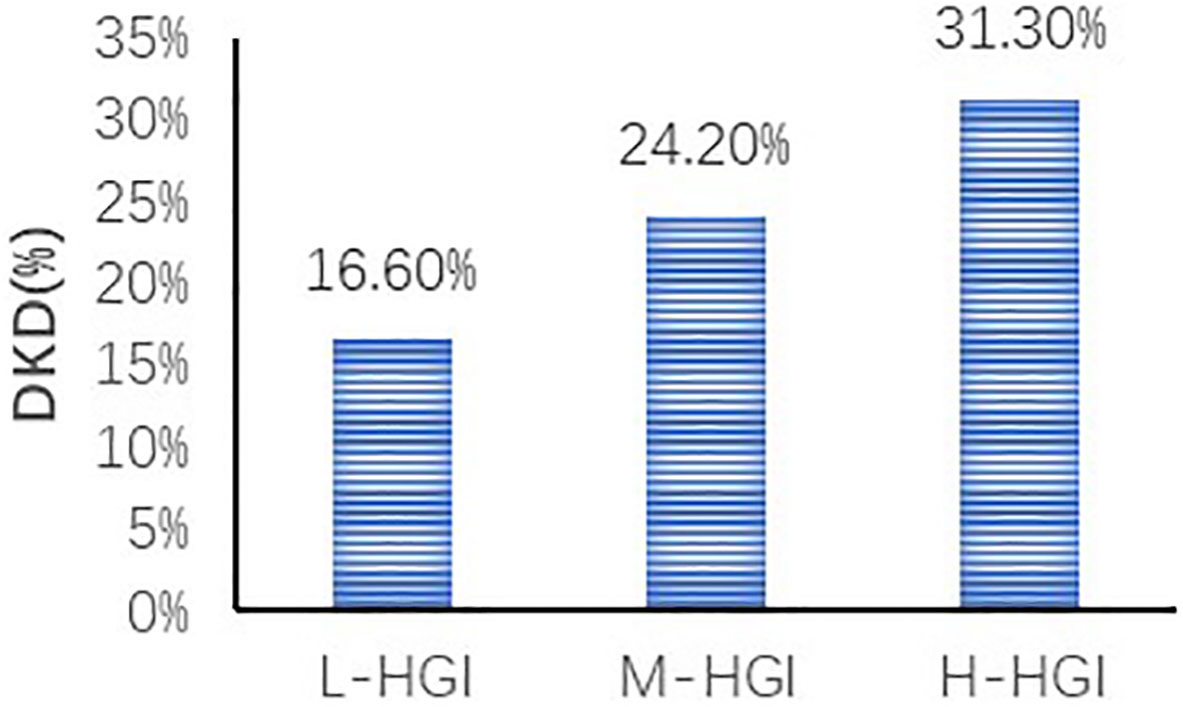
Comparison of prevalence of DKD in different HGI groups. We found an increase in the prevalence of DKD in three groups. The difference was statistically significant (P=0.000). Correlation analysis revealed that with the increase of HGI level the prevalence of DKD was increasing (r= 0.140, P=0.000).

There were significant differences in age (P=0.033), T2DM duration (P=0.005), SBP (P=0.003), HbA1c (P=0.000), FPG (P=0.032), 2h-PPG (P=0.000), FCP (P=0.000), 2h-CP (P=0.000), TC (P=0.003), LDL-C (P=0.000), sCr (P=0.001), eGFR (P=0.000) among the three groups. There were no significant differences in sex (P=0.299), DBP (P=0.058), BMI (P=0.274), TG (P=0.932), HDL-C (P=0.327), UA (P=0.089), UACR (P=0.111). The prevalence of DKD among the three groups was 16.60%, 24.20% and 31.30%. The difference was statistically significant (P=0.000). Mantel-Haenszel chi-square test showed that there was a linear relationship between HGI and DKD (x^2 =^ 31.817, P=0.000). Pearson correlation analysis showed that with the increase of HGI level the prevalence of DKD was increasing (r= 0.140, P=0.000). (**Table 1**, **Figure 2**)

**Table 1:** Comparison of general data and biochemical index results in each group.

Variable	L-HGI (*n*=541)	M-HGI (*n*=541)	H-HGI (*n*=540)	*F*(*X* ^2^)	*p*
Sex [n(M/F)]	*353/188*	*330/211*	*333/207*	2.416	0.299
Age (yeas)	55.70 ± 12.94	56.86 ± 13.40	54.73 ± 14.00	3.415	0.033
T2DM Duration (yeas)	9.00 ± 7.73	10.17 ± 7.69	8.74 ± 7.69	5.307	0.005
SBP (mmHg)	130.90 ± 16.23	134.53 ± 17.55	132.56 ± 18.06	5.961	0.003
DBP (mmHg)	78.10 ± 11.13	79.31 ± 10.36	79.60 ± 11.30	2.858	0.058
BMI (Kg/m^2^)	25.78 ± 3.73	26.14 ± 3.65	26.00 ± 3.65	1.296	0.274
FBG (mmol/L)	8.53 ± 3.27	8.91 ± 3.29	9.01 ± 3.00	3.435	0.032
PBG (mmol/L)	11.66 ± 4.19	12.76 ± 4.38	13.47 ± 4.68	23.119	0.000
HbA1c (%)	6.99 ± 1.34	8.46 ± 1.28	10.43 ± 1.40	937.672	0.000
FCP (ng/ml)	2.66 ± 1.31	2.43 ± 1.31	2.14 ± 1.38	19.637	0.000
2h-CP (ng/ml)	6.81 ± 4.07	5.66 ± 3.36	4.65 ± 3.60	43.579	0.000
TC (mmol/L)	4.23 ± 1.04	4.35 ± 1.15	4.47 ± 1.16	5.976	0.003
TG (mmol/L)	2.08 ± 2.00	2.05 ± 1.60	2.09 ± 1.62	0.080	0.923
HDL-C (mmol/L)	1.02 ± 0.27	1.00 ± 0.27	1.00 ± 0.26	1.119	0.327
LDL-C (mmol/L)	2.43 ± 0.83	2.56 ± 0.90	2.64 ± 0.92	8.059	0.000
UA (umol/L)	345.30 ± 91.16	349.17 ± 96.78	336.85 ± 94.44	2.421	0.089
Crea (umol/L)	68.77 ± 19.49	71.87 ± 35.99	65.66 ± 17.94	7.835	0.000
eGFR (ml/min/1.73m^2^)	97.42 ± 18.58	95.44 ± 21.78	100.28 ± 18.69	8.196	0.000
UACR (mg/g)	60.79 ± 280.66	103.63 ± 422.70	96.65 ± 363.17	2.202	0.111
DKD (%)	90 (16.6)	131 (24.2)	169 (31.3)	31.817	0.000

### 3.2 Binary logistic regression analysis of the relationship between DKD and related factors and HGI risk analysis in T2DM

To investigate the potential interactions affecting the prevalence of DKD in T2DM, binary logistic regression analysis was performed as shown in **Table 2**. It was shown that age, T2DM duration, SBP, FCP, TC, eGFR, HGI were risk factors for DKD. Furthermore, in our study, HGI was found having a strong link with the incidence of DKD. Univariate logistic regression analysis showed that compared with L-HGI group, the risk of DKD in H-HGI group was significantly increased (OR: 2.283, 95% CI: 1.708~ 3.052). After adjusting for age, T2DM duration, SBP, TC, FCP, eGFR, the risk of DKD in H-HGI group was 2.66 times than that in L-HGI group. (**Table 3**)

**Table 2:** Binary logistic regression analysis of risk factors for DKD in T2DM.

Variables	DKD in T2DM		
	*β*st	OR (95% CI)	*P*
Age (yeas)	-0.017	0.984 (0.971-0.997)	0.015
T2DM Duration (yeas)	0.042	1.043 (1.024-1.063)	0.000
SBP (mmHg)	0.023	1.023 (1.016-1.030)	0.000
FCP (ng/ml)	0.164	1.179 (1.070-1.299)	0.001
TC (mmol/L)	0.117	1.124 (1.007-1.255)	0.037
eGFR (ml/min/1.73m^2^)	-0.024	0.976 (0.968-0.984)	0.000
HGI	0.212	1.236 (1.135-1.346)	0.000

**Table 3:** Logistic regression analysis of the relationship between HGI and DKD in T2DM.

HGI level	Unadjusted model	*P^1^*	Multivariate model^a^	*P^2^*
	*OR^1^ (95%CI)*		*OR^2^ (95%CI)*	
L-HGI	1.000 (ref)	0.000	1.000 (ref)	0.000
M-HGI	1.6019 (1.186-2.161)	0.002	1.414 (1.023-1.995)	0.036
H-HGI	2.283 (1.708-3.052)	0.000	2.660 (1.935-3.657)	0.000

a Adjusted for age, T2DM duration, SBP, TC, FCP, eGFR.

### 3.3 Construction of a risk score for DKD

**Figure 3:**
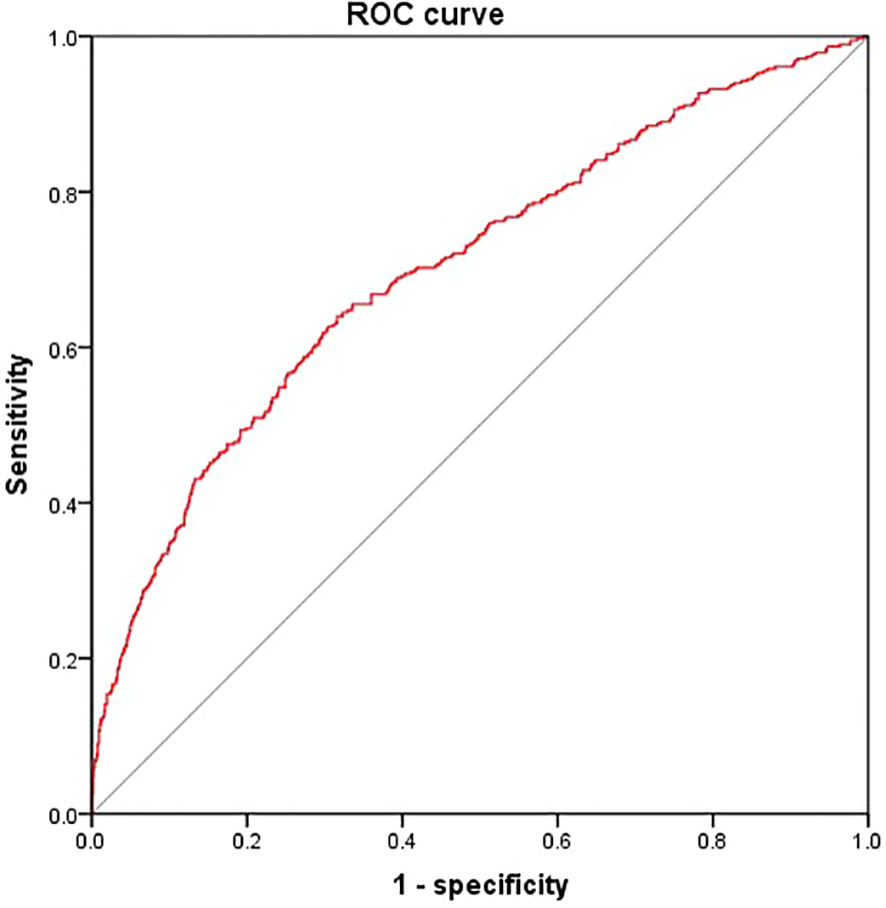
Receiver operating characteristics curves of DKD risk score. We put HGI, age, T2DM duration, SBP, TC, FCP and eGFR into the model The area below the ROC curve of this model was 0.702 (95%*CI*: 0.671 - 0.734). The sensitivity and specificity corresponding to the maximum Youden index were 0.640% and 0.649% respectively.

According to the results, the variables such as HGI, age, T2DM duration, SBP, TC, FCP, eGFR were the key risk factors (P < 0.05). We put them into the model, which determined the risk of DKD [DKD risk score =0.212* HGI + 0.042* T2DM duration (year) + 0.023* SBP (mmHg) + 0.164* FCP (ng/ml)– 0.017* age (year)- 0.024* eGFR (ml/min/1.73m^2^)- 2.301]. The area below the receiver operating characteristics (ROC) curve of this model was 0.702 (95%*CI*: 0.671 - 0.734), which showed good discrimination ability. The sensitivity and specificity corresponding to the maximum Youden index were 0.640% and 0.649%, respectively (**Figure 3**).

## 4 Discussion

In this Cross- Sectional study of Chinese adults with T2DM, we found an association between HGI and incident DKD. A dramatic increase in DKD incidence was observed among subjects with higher values of HGI. Our findings suggested that HGI may be a useful predictor of incident DKD among patients with T2DM.

HbA1c value is generally considered the gold standard method for evaluating glycemic control. However, in three large randomized controlled clinical trials (18–20), namely, the Action to Control Cardiovascular Risk in Patients with Diabetes (ACCORD), ADVANCE and the Veterans Affairs Diabetes Trial (VADT), intensive glycemic control in patients with T2DM did not benefit large blood vessels. Especially in the ACCORD trial, as compared with standard therapy, intensive therapy to target normal HbA1c levels for 3.5 years increased mortality and did not significantly reduce major cardiovascular events (21). Accordingly, Sheng et al. (22) proposed a hypothesis that HbA1c variability may be related to all-cause mortality of intensive therapy, and conducted a *post hoc* analysis of the ACCORD trial. This study showed that long-term follow-up HbA1c variability was a strong predictor of all-cause mortality. Thus, HbA1c is not a one-size-fits-all indicator of blood glucose control. This phenomenon might be attributed to various biological factors, including genetic predisposition, erythrocyte turnover rates, intracellular glucose concentrations, intracellular or extracellular pH, lipid peroxides, inorganic phosphates, hemoglobin oxygenation status, cellular redox status, and the activity of non-enzymatic protein glycation (9, 23).

Increasing evidence is supporting the role of glucose variability (GV) in the development of diabetic complications (24). Studies in recent years (25) have shown that HbA1c levels vary greatly among individuals, and some patients may have high or low HbA1c levels inconsistent with blood glucose control levels, which brings some difficulties to clinical prognosis assessment based on this indicator. HGI can identify people with HbA1c levels that are higher or lower than average compared to other people with the same blood glucose concentration (8, 9). Biological sources of HGI variation include genetic and environmental factors that affect person-to-person variation in HbA1c or blood glucose. In our study, we also found that only 40.5% of HbA1c variation can be explained by FPG. In addition, Sabanayagam C. et al. (26) designed a study to determine whether the relationship of HbA1c to diabetic microvascular complications showed any natural thresholds that could be useful in diagnosing diabetes. There data supported use of an HbA1c cut-off point of between 6.6 and 7.0% in diagnosing diabetes. Any retinopathy, CKD, albuminuria and peripheral neuropathy were less well detected at these cut-off points. Our study also suggested that the incidence of DKD in L-HGI group [HbA1c (6.99 ± 1.34) %] is lowest, similar to previous studies.

Before 2015, several studies shown a positive association between GV and diabetic complications, both macrovascular and microvascular (27). Since 2015, new evidence has also emerged in support of GV as an independent risk factor for total mortality and death due to cardiovascular disease in both type 1 and type 2 diabetes (11, 28–32). R. J. McCarter et al. (12) concluded that individual biological variation in HbA1c, which is distinct from that attributable to mean blood glucose (MBG), was evident among type 1 diabetic patients in DCCT and was a strong predictor of risk for diabetic complications. At 7 years’ follow-up, patients in H-HGI had three times greater risk of retinopathy (30 *vs*. 9%, P < 0.001) and six times greater risk of nephropathy (6 *vs*.1%, P < 0.001) compared with the L-HGI. The individual variation in HbA1c observed in DCCT was attributable to biological variation and not measurement error. Evidence of a link between biological variation in HbA1c and microvascular complications in DCCT suggested that factors responsible for biological variation in nonenzymatic HbA1c may also influence individual susceptibility to diabetic complications. Chih-Hung Lin et al. (14) found that a high HGI predicted rapid renal function decline without or with a resultant eGFR < 60 ml/min/1.73m^2^, but not onset of macroalbuminuria followed for a median of 7.3 years. Thus, HGI independently predicted renal function deterioration in patients with T2DM and a low CKD risk. In patients with T2DM, HbA1c variability affects CKD more than average HbA1c (33). Even in nondiabetic individuals, studies have reported the effect on HGI and kidney dysfunction and CKD among the non-diabetic individuals and the adults with hypoglycemic drug naive prediabetes and diabetes. Teresa Vanessa Fiorentino et al. concluded that HGI may be a useful tool to identify nondiabetic individuals with an increased risk of having kidney dysfunction (34). Wonjin Kim et al. found an association between HGI and incident CKD. High HGI was associated with an increased risk of incident CKD. Regardless of HbA1c value, subjects with higher values of HGI were at a higher risk of incident CKD during the 10-year follow-up period (35).

In the ACCORD population, baseline age, BMI, SBP, DBP, fasting serum glucose, TC and SCr had significant difference compared with low HbA1c variability, while sex and LDL-C had not (22). Most of these results have been confirmed in our study but of BMI and LDL-C, suggesting that the influencing factor of HGI in T2DM is complex and that the causes are multifactorial. In addition, further logistic regression analysis showed that the age, T2DM duration, SBP, TC, FCP, 2h-CP, eGFR and HGI were the key risk factors of DKD in this study. The risk of DKD in T2DM patients with high HGI levels is 2.283 times higher than those with low HGI levels. Adjusted to multiple factors, this trend still remained significant (OR: 2.660, 95%CI: 1.935~ 3.657). Furthermore, we constructed a risk score based on HGI to predict a person’s risk of DKD, which could be useful for the clinician.

At present, the mechanism of HGI on DKD in T2DM is still unclear. The possible mechanisms involve the following aspects: (1) Fluctuation of blood glucose leads to increased oxidative stress, production of inflammatory cytokines and endothelial dysfunction (36, 37). Compared with persistent hyperglycemia, islet β-cell dysfunction and apoptosis increased significantly in the state of blood glucose fluctuation which leading to decrease of insulin secretion (38). (2) Fluctuated blood glucose deteriorated the progression of DKD by increasing the blood urea nitrogen and sCr, decreasing creatinine clearance, and accelerating renal ultrastructural injury. This adverse result was probably due to its promoting oxidative stress activity and the p-AKT signaling pathway inhibition, which activated its downstream proteins, resulting in severe renal injury (39). (3) One possible explanation is that even periods of sustained hyperglycemia are “remembered,” thus conferring an increased risk of microvascular complications (40, 41), hence, the detrimental effect of HbA1c variability may be mediated through the same mechanism underlying the “metabolic memory” phenomenon, including oxidative stress. (4) Because the risk of microvascular complications increases exponentially as HbA1c rises (42), subjects with higher HbA1c variability would “accumulate” a surplus of risk in the periods spent at the upper end of their HbA1c range. This hypothesis might be indirectly supported by GIUSEPPE PENNO’s observation that the effect of HbA1c variability is a statistically significant effect in the higher quartile of HbA1c-SD (33).

## 5 Conclusion

To sum up, HGI may be a reference index for blood glucose control in T2DM patients and could predict the risk of DKD. This study brings important enlightenment for daily diabetes management, that is, diabetes patients should take into account the variability of HbA1c while controlling blood glucose or HbA1c levels. “Beyond HbA1c” is an important concept of diabetes diagnosis and treatment at present (43). Blood glucose, glycosylated albumin and HbA1c variability are important factors for blood glucose control and long-term prognosis. In order to reduce the risk of all-cause death in diabetic patients, measures should be taken as soon as possible to incorporate HbA1c variability into the management objectives of diabetic patients.

This study also has some limitations. As the analysis was conducted on the basis of cross section, the existing database cannot collect relevant data at the time of diagnosis of diabetes. Unfortunately, previous studies have not reported it yet. Next, we will focus on collecting data of new-onset T2DM, and make further exploration on the impact of HGI on DKD. In addition, the subjects have not been followed up to determine the relationship between HGI and DKD in the existing cross-sectional study. However, according to the current research results, HGI has stable disease prediction value. Furthermore, high-quality and large sample prospective cohort studies and randomized controlled clinical trials and even cytological studies will be carried out to clarify the mechanism of HGI and the predictive value of HGI on DKD, and to develop a personalized HbA1c variability control target, so as to provide new reference indicators for clinical diabetic blood glucose control and reduce the occurrence of complications.

## Data availability statement

The raw data supporting the conclusions of this article will be made available by the authors, without undue reservation.

## Ethics statement

The studies involving human participants were reviewed and approved by Biomedical Ethics Committee of Peking University International Hospital. Written informed consent for participation was not required for this study in accordance with the national legislation and the institutional requirements.

## Author contributions

XZhan, SX, XZhao and JD made substantial contributions to the conception and design of the study. SX and JD collected the data. SX and XZhao analyzed the data. SX and XZhan drafted the manuscript. All authors contributed to the article and approved the submitted version.
