# Corrigendum: Association between hemoglobin glycation index and diabetic kidney disease in type 2 diabetes mellitus in China: a cross-sectional inpatient study

**DOI:** 10.3389/fendo.2023.1199643

**Published:** 2023-05-19

**Authors:** Sixu Xin, Xin Zhao, Jiaxiang Ding, Xiaomei Zhang

**Affiliations:** ^1^ Department of Endocrinology, Peking University International Hospital, Beijing, China; ^2^ Department of Nephrology, Peking University International Hospital, Beijing, China

**Keywords:** diabetes mellitus, type 2, hemoglobin glycation index, diabetic kidney disease, complication

In the published article, there were errors in the list of author affiliations. Instead of “^1^Department of Endocrinology, International Hospital, Peking University, Beijing, China, ^2^Department of Nephrology, International Hospital, Peking University, Beijing, China]”, it should be “^1^Department of Endocrinology, Peking University International Hospital, Beijing, China, ^2^Department of Nephrology, Peking University International Hospital, Beijing, China”.

The authors apologize for this error and state that this does not change the scientific conclusions of the article in any way. The original article has been updated.

